# Virtual Screening and Molecular Dynamics of Cytokine–Drug Complexes for Atherosclerosis Therapy

**DOI:** 10.3390/ijms26072931

**Published:** 2025-03-24

**Authors:** María Angélica Rodríguez-Fernández, Fabiola Estefanía Tristán-Flores, Diana Casique-Aguirre, María de la Luz Xochilt Negrete-Rodríguez, Juan Antonio Cervantes-Montelongo, Eloy Conde-Barajas, Gerardo Acosta-García, Guillermo Antonio Silva-Martínez

**Affiliations:** 1 Posgrado de Ingeniería Bioquímica, Tecnológico Nacional de México/IT de Celaya, Celaya 38010, Guanajuato, Mexico; d2403022@itcelaya.edu.mx (M.A.R.-F.); fabiola.tristan@itcelaya.edu.mx (F.E.T.-F.); xochilt.negrete@itcelaya.edu.mx (M.d.l.L.X.N.-R.); eloy.conde@itcelaya.edu.mx (E.C.-B.); gerardo.acosta@itcelaya.edu.mx (G.A.-G.); 2 Departamento de Ciencias Básicas, Tecnológico Nacional de México/IT de Celaya, Celaya 38010, Guanajuato, Mexico; 3 Laboratorio de Citómica del Cáncer Infantil, Centro de Investigación Biomédica de Oriente, Instituto Mexicano del Seguro Social, Delegación Puebla, Puebla 06600, Mexico; diana.casique@secihti.mx; 4 Secretaría de Ciencia, Humanidades, Tecnología e Innovación (SECIHTI), Ciudad de México 03940, Mexico; 5 Departamento de Ingeniería Bioquímica y Ambiental, Tecnológico Nacional de México/IT de Celaya, Celaya 38010, Guanajuato, Mexico; jcervantesm@udec.edu.mx; 6 Escuela de Medicina, Universidad de Celaya, Celaya 38080, Guanajuato, Mexico

**Keywords:** atherosclerosis, virtual screening, pro-atherogenic cytokines, foam cells, cytokine-targeted therapy, molecular dynamics

## Abstract

Cardiovascular disease remains the leading global cause of mortality, largely driven by atherosclerosis, a chronic inflammatory condition characterized by lipid accumulation and immune-cell infiltration in arterial walls. Macrophages play a central role by forming foam cells and secreting pro-atherogenic cytokines, such as TNF-α, IFN-γ, and IL-1β, which destabilize atherosclerotic plaques, expanding the lipid core and increasing the risk of thrombosis and ischemia. Despite the significant health burden of subclinical atherosclerosis, few targeted therapies exist. Current treatments, including monoclonal antibodies, are limited by high costs and immunosuppressive side effects, underscoring the urgent need for alternative therapeutic strategies. In this study, we employed in silico drug repositioning to identify multitarget inhibitors against TNF-α, IFN-γ, and IL-1β, leveraging a virtual screening of 2750 FDA-approved drugs followed by molecular dynamics simulations to assess the stability of selected cytokine–ligand complexes. This computational approach provides structural insights into potential inhibitors. Additionally, we highlight nutraceutical options, such as fatty acids (oleic, linoleic and eicosapentaenoic acid), which exhibited strong and stable interactions with key cytokine targets. Our study suggests that these bioactive compounds could serve as effective new therapeutic approaches for atherosclerosis.

## 1. Introduction

Atherosclerosis is a chronic inflammatory disease characterized by the accumulation of lipid particles in the arterial walls and the recruitment of inflammatory and immune cells, including macrophages [1]. This gradual process often leads to severe complications such as myocardial infarction and stroke. Globally, ischemic heart disease, driven largely by atherosclerosis, remains the leading cause of mortality, responsible for approximately 9 million deaths annually [2,3]. Risk factors for atherosclerosis include hypercholesterolemia, hypertension, diabetes mellitus, kidney disease, and smoking, among others [4]. Despite medical advancements in certain regions, atherosclerosis continues to be the foremost contributor to global mortality [5].

During atherosclerosis, macrophages absorb modified cholesterol, resulting in macrophages loaded with lipids and thus producing foam cells, which, having a migratory capacity, become trapped in the plaque, where they die and form a necrotic nucleus [6]. Macrophages infiltrate the arterial intima, taking up excess lipids and forming foam cells [7]. This process that results in necroptosis is triggered by various molecule interactions, including various types of cytokines [8].

Therapies targeting foam cells in atherosclerotic plaques are in the early stages due to limited biological knowledge [9]. Currently used treatments such as monoclonal antibodies, including Infliximab, Adalimumab, Etanercept, Certolizumab, and Golimumab, have several side effects [10]. These include eliciting an antigen–antibody response against self-antigens, weakening the body’s defensive system, and their high cost.

Cytokines are small proteins that mediate communication between neighboring cells in lymphoid organs or inflamed tissues [11]. Numerous studies have shown that specific cytokines participate in different stages of immune-cell activation, regulate internal and external lipid flow, and are essential chemical mediators in various pathophysiological processes, such as intercellular signal transduction [12]. The biological effects of pro-inflammatory cytokines, such as tumor necrosis factor-α (TNF-α), interleukin-1β (IL-1β), and interferon-γ (IFN-γ), may account for their pro-atherogenic activity include apoptosis and foam-cell formation [13].

TNF-α is a pro-inflammatory cytokine with pleiotropic effects on immune cells, participating in the inflammatory response in atherosclerosis [14]. It also contributes to the pathogenesis of other diseases such as rheumatoid arthritis [15], Crohn’s disease [16], and ulcerative colitis [17]. During the progression of atherosclerosis, TNF-α mediates its inflammatory response and physiological functions by binding to its receptors, TNFR1 in its secreted form and TNFR2 in its membrane form [18]. TNF-α is associated with the propagation of heart failures such as myocardial hypertrophy and ischemia [19] and has a critical role in intermediary metabolism; therefore, its deregulation could contribute to the development of cardiovascular diseases. Although numerous small-molecule inhibitors against TNF-α have been identified, no orally active drug has yet been reported urging an urgent need for a small molecule anti-TNF-α drug [20].

IL-1β is a cytokine that regulates the immune system [21]. Overproduction of this molecule plays a critical role in diseases such as rheumatoid arthritis, inflammatory bowel disease, osteoarthritis, vascular disease, multiple sclerosis, neuropathic pain and Alzheimer’s disease [22]. It has been reported that the levels of IL-1 β protein and mRNA increase significantly in atherosclerosis patients and are positively correlated with disease severity compared to normal subjects [23]. Studies have demonstrated that IL-1 β, when secreted, promotes the formation of macrophage foam cells through autocrine signaling. Secreted IL-1β serves as an autocrine function for promoting macrophage foam-cell formation in THP-1 macrophage cell models [24].

IFN-γ is a pro-inflammatory cytokine capable of inducing the de-expression of several genes expressed in macrophages [25]. An increase in atherosclerotic lesions has been observed in apolipoprotein E mice injected with recombinant IFN-γ [26]. IFN-γ also promotes foam-cell apoptosis, expulsing its contents into the intima, and therefore contributing to a lipid-rich necrotic core and extracellular matrix [27].

It has been suggested that single-cytokine inhibition is not likely to be a successful therapeutic strategy [28]; therefore, the proposed strategy is to identify a small molecule through drug repositioning that targets multiple cytokines simultaneously.

Computational drug repositioning, supported by artificial intelligence (AI)-driven tools like AlphaFold, offers a promising approach to accelerating the discovery of cytokine inhibitors. This method enables the structural prediction of protein–ligand interactions, allowing for the identification of FDA-approved drugs with potential multitarget activity against TNF-α, IFN-γ, and IL-1β. In silico approaches, such as molecular docking and molecular dynamics (MD) simulations, provide valuable insights into ligand binding stability and bioactivity, significantly reducing the time and cost associated with conventional drug-discovery pipelines [29]. This innovation accelerates drug discovery by providing molecular insights into protein–ligand interactions, facilitating the identification of therapeutic targets, and streamlining the development of novel treatments for complex diseases like atherosclerosis. In this study, we applied virtual screening to identify multitarget inhibitors among 2750 FDA-approved drugs, followed by MD simulations to assess the stability and feasibility of selected ligand–cytokine complexes. Additionally, we highlight nutraceutical compounds, particularly fatty acids, which exhibited stable interactions with pro-atherogenic cytokines, supporting their potential role in atherosclerosis therapy. Our findings contribute to the development of novel, cost-effective, and safer therapeutic options, bridging computational drug discovery with clinical applications.

## 2. Results

### 2.1. Structural Analysis of TNF-α, Il-1β and IFN-γ

**Figure 1:**
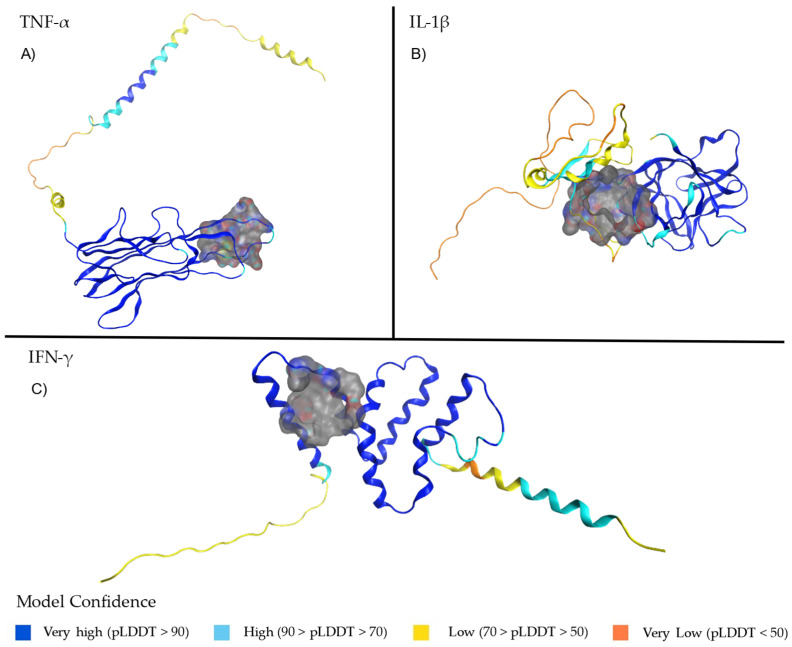
Binding sites selected for molecular docking in the three target cytokines. (**A**) Binding site identified in TNF-α; (**B**) binding site identified in IL-1β; (**C**) binding site identified in IFN-γ. These structures represent the regions selected for molecular interaction assays. The reliability of the predicted regions in the model is based on the predicted local-distance difference test (pLDDT) scores.

AlphaFold-predicted structures often exhibit extensive unstructured regions, which are typically absent in crystallographic models. This structures often differ from experimental models, particularly in loop flexibility and disordered regions, which may lead to artificial fluctuations in MD simulations. Unlike crystallographic data, AlphaFold can overestimate confidence in flexible regions, affecting structural stability assessments [30]. These regions introduce inherent structural flexibility, impacting the accuracy of molecular simulations. Potential ligand-binding sites for TNF-α (Figure 1A), IL-1β (Figure 1B), and IFN-γ (Figure 1C) structures were identified by selecting cavities containing residues involved in the activity of each protein. These binding sites are detailed in Table 1. TNF-α predicted structures include residues that are located in the E–F loop, and we selected a site located at the part of the monomer that corresponds to an important part of pore formed through the center of the trimer [31,32], with the residues 178-180 being of particular interest, which form the core epitope [33]. The confidence levels of the structural model of each protein are described and represented in a color scheme in Figure 1 [34].

The potential binding site selected for the IFN-γ structure comprises the residue LEU136, whose C-terminal truncation was previously shown to be important, along with GLU135, in the reduction in JAK-STAT1 signaling and proinflammatory macrophage activation [35]. For IL-1β, the selected site corresponded to a pocket comprising the N-terminal residue ARG120, which plays an important role in stabilization of the tertiary structure of the protein and receptor-binding domain [36] as well as SER43, TYR68, and PRO87 residues that correspond to the binding site previously reported for other related small molecules [37]. The modeled structure of IL-1β comprises TYR68 and TRP120 residues that undergo a proteolytic cleavage by Caspase-1 in the IL-1β precursor, Pro-IL-1β. Pro-IL-1β retains a stable core region at the C-terminus, which eventually becomes the functional mature protein (IL-1 β). This highly protected region in pro-IL-1β corresponds to a fundamental part in the structure and biological activity of mature IL-1β [38]. The selected potential binding sites for TNF-α, IL1-β and IFN-γ described in Table 2 were key residues, and are highlighted in blue.

### 2.2. Virtual Screening and Molecular Docking of Active Compounds

The in-house database was analyzed for the top 10 ligands based on their S score, binding frequency, which drug binds to the receptor site, and the types of interactions, preferably with the hydrogen bond of the selected ligand. In molecular docking, the S score represents the predicted binding affinity of a ligand to a target protein. A lower S score indicates a more favorable binding pose, suggesting stronger interactions between the ligand and the protein [39]. The S score range in TNF-α spans from −4.31 to −6.77, whereas the range for IL-1β spans from −5.52 to −9.13 and the range for IFN-γ spans from −8.54 to −11.40 (Table 3).

**Figure 2:**
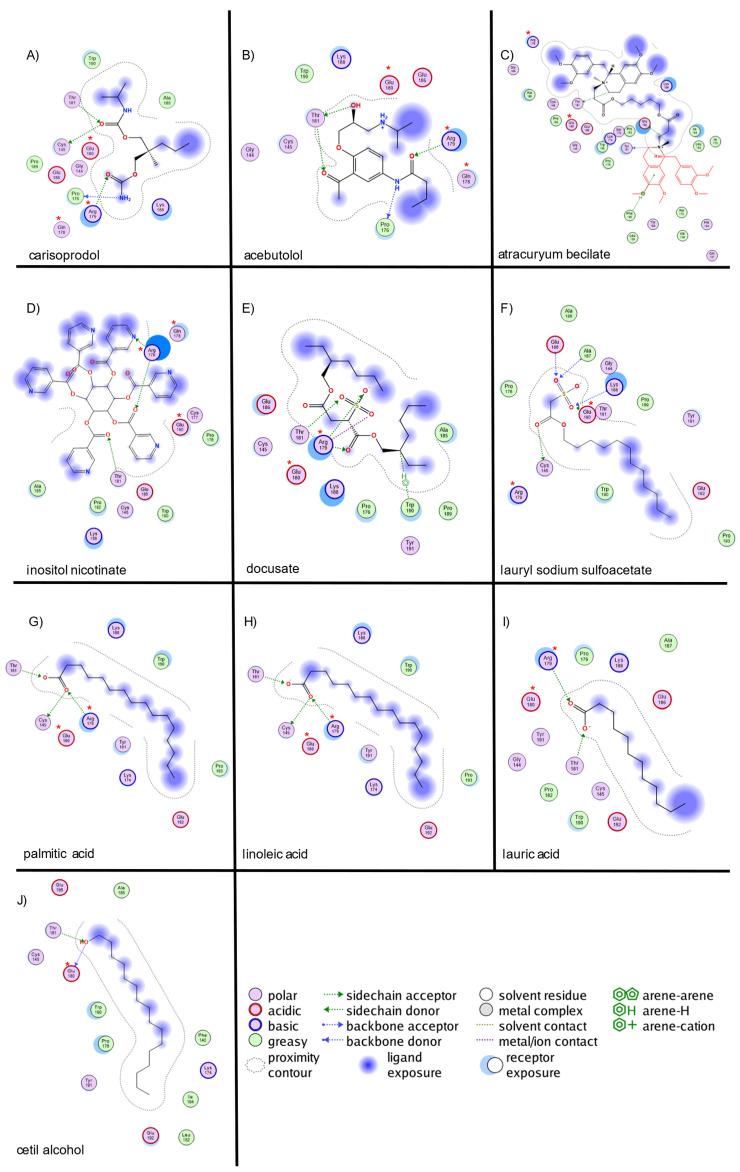
Molecular interaction map of top 10 ligands with TNF-α site. The blue arrows indicate the structural hydrogen bridge bonds, and the green arrows are the hydrogen bridge bonds with the side chain. TNF-α interacts with (**A**)—carisoprodol, (**B**)—acebutolol, (**C**)—atracutyum becilate, (**D**)—inositol nicotinate, (**E**)—docusate, (**F**)—lauryl sodiumsulfoacetate, (**G**)—palmitic acid, (**H**)—linoleic acid, (**I**)—lauric acid, and (**J**)—celtil alchohol. (*) Indicates key structural residues.

For TNF-α (Figure 2), carisoprodol exhibited hydrogen-bond interactions with CYS145, PRO176, THR181, and ARG179 (two interactions). Acebutolol formed hydrogen bonds with PRO176, THR181 (donor and acceptor), and ARG179. The atracurium besilate showed hydrogen bonding with THR181, TYR191, and GLU192, along with a π-interaction with PHE140. Inositol nicotinate interacted via hydrogen bonds with ARG179 (two interactions) and THR181. Docusate formed hydrogen bonds with ARG179 (two interactions) and THR181, along with ionic bonds with ARG179 (two interactions) and a π-interaction with TRP190. Lauryl sodium sulfoacetate exhibited hydrogen bonding with GLU180 (two interactions) and THR181. Palmitic acid formed hydrogen bonds with CYS145, THR181, and ARG179, and ionic bonds with ARG179 (two interactions). Linoleic acid showed hydrogen bonding with ARG179 (two interactions) and THR181, along with ionic interactions with ARG179 (two interactions). Lauric acid interacted via hydrogen bonds with THR181 and ARG179 (two interactions), with additional ionic bonds at ARG179 (two interactions). Cetil alcohol exhibited hydrogen bonds with GLU180 and THR181.

**Figure 3:**
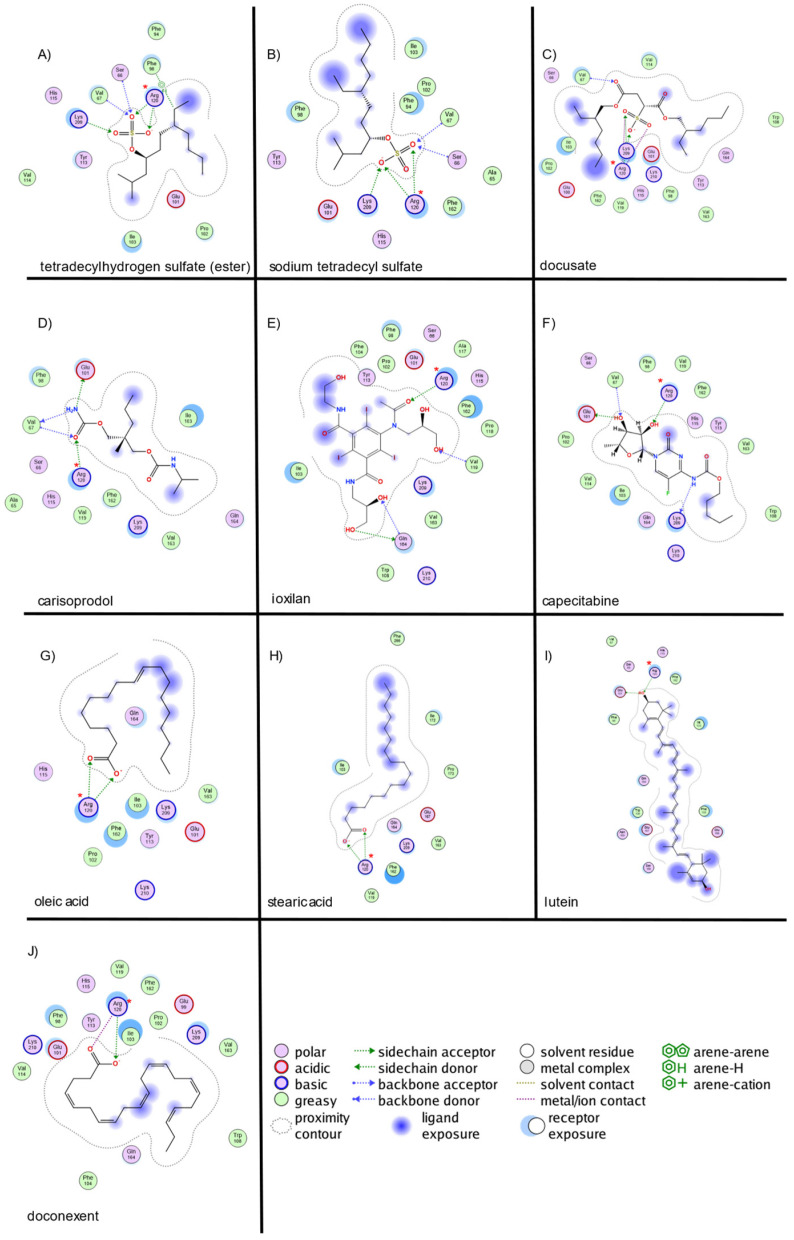
Molecular interaction of top 10 ligands with IL-1β site. The blue arrows indicate the structural hydrogen bridge bonds, and the green arrows are the hydrogen bridge bonds with the side chain. IL-1β interacts with (**A**)—tetradecyl hydrogen sulfate (ester), (**B**)—sodium tetradecyl sulfate, (**C**)—docusate, (**D**)—carisoprodol, (**E**)—ioxilab, (**F**)—capecitabine, (**G**)—oleic acid, (**H**)—stearic acid, (**I**)—luteion, and (**J**)—doconexent. (*) Indicates key structural residues.

For IL-1β (Figure 3), tetradecyl hydrogensulfate (ester) exhibited hydrogen-bond interactions with LYS209, SER66, VAL67, and ARG120 (2 interactions), along with ionic interactions at ARG120 (4 interactions) and a π-bond with PHE98. Sodium tetradecyl sulfate formed hydrogen bonds with SER66, VAL67, ARG120 (2 interactions), and LYS209, and ionic interactions with ARG120 (4 interactions). Docusate displayed hydrogen bonds with LYS209, ARG120 (2 interactions), and VAL67, along with ionic interactions at ARG120 (6 interactions). Carisoprodol interacted via hydrogen bonds with VAL67, GLU101, and ARG120. Ioxilan established hydrogen bonds with GLN164 (donor/acceptor), ARG120, and VAL119. Capecitabine exhibited hydrogen bonds with GLU101, LYS209, ARG120, and VAL67, along with π-interactions at two residues. Oleic acid displayed hydrogen bonds with ARG120 (3 interactions) and ionic interactions with ARG120 (4 interactions). Stearic acid formed hydrogen bonds with ARG120 (3 interactions) and LYS209, along with ionic interactions with ARG120 (4 interactions). Lutein interacted with GLU101 and ARG120 via hydrogen bonding. Doconexent displayed hydrogen bonds with ARG120 (2 interactions) and ionic interactions with ARG120 (5 interactions).

**Figure 4:**
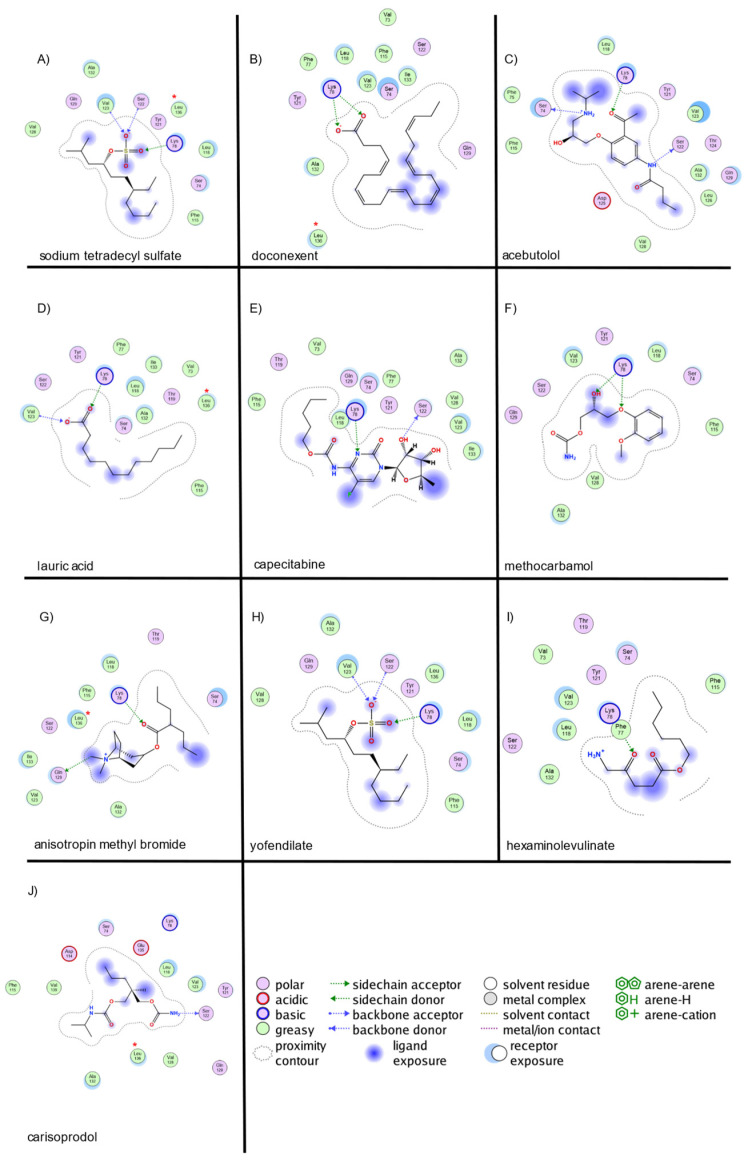
Molecular interaction map of top 10 ligands with IFN-γ site. The blue arrows indicate the structural hydrogen bridge bonds, and the green arrows are the hydrogen bridge bonds with the side chain. IFN-γ interacts with (**A**)—sodium tetradecyl sulfate, (**B**)—doconexent, (**C**)—acebutolol, (**D**)—lauric acid, (**E**)—capecitabine, (**F**)—methocarbamol, (**G**)—anisotropoinmethyl bromide, (**H**)—yofendilate, (**I**)—hexaminolevulinate, and (**J**)—carisoprodol. (*) Indicates key structural residues.

For IFN-γ (Figure 4), sodium tetradecyl sulfate exhibited hydrogen bond interactions with LYS78, SER122, and VAL123, along with ionic interactions at LYS78. Doconexent formed hydrogen bonds with LYS78 (2 interactions) and ionic interactions with LYS78 (2 interactions). Acebutolol interacted via hydrogen bonds with SER122, SER74, and LYS78. Lauric acid displayed hydrogen-bond interactions with VAL123 and LYS78, with ionic interactions at LYS78. Capecitabine exhibited hydrogen bonding with SER122 and LYS78. Methocarbamol interacted with LYS78 (two hydrogen bonds). Anisotropin methyl bromide formed hydrogen bonds with GLN129 and LYS78. Yofendilate presented a hydrogen bond with LYS78 and a π-interaction with VAL123. Hexaminolevulinate exhibited hydrogen bonding with LYS78 and a π-interaction. Carisoprodol interacted with LYS78 via hydrogen bonding.

The strongest interactions for TNF-α were observed with PRO176, ARG179, and THR181, consistent with previously reported key binding sites. For IFN-γ, interactions predominantly involved LYS78, SER122, VAL123, and GLN129, corresponding to the predicted binding site. In IL-1β, the most significant interactions were identified with ARG120, SER66, VAL67, and LYS209, aligning with previously reported small-molecule binding sites.

### 2.3. Pharmacokinetic Predicted Profiles of Selected Ligands and Molecular Dynamics Simulations

MD simulations were performed to evaluate the stability, binding interactions, and conformational behavior of doconexent (docosahexaenoic acid, DHA), linoleic acid, and oleic acid in complex with TNF-α, IL-1β, and IFN-γ. These fatty acids were selected based on their high docking scores, strong interactions with key residues, and biological relevance, highlighting their potential as therapeutic agents for atherosclerosis [40]. They possess a notable binding affinities, biological relevance, and comparative advantages. The docking results revealed that DHA had one of the highest scores (−9.66, with IFN-γ), while linoleic acid and oleic acid demonstrated interactions with the residues ARG120, THR181, and ARG179, essential for the protein’s functional regions. Biologically, these fatty acids are well known for their anti-inflammatory and cardioprotective properties, making them particularly suitable for mitigating atherosclerosis progression [41,42,43]. Additionally, their natural occurrence and metabolic compatibility offer a distinct advantage over synthetic compounds, ensuring greater safety and efficacy in modulating lipid metabolism and cellular signaling pathways. Focusing on these fatty acids aligns computational predictions with their therapeutic potential, establishing a robust foundation for the development of targeted treatments for atherosclerosis.

Before MD simulations, we perform SwissADME analysis on linoleic, oleic, and docosahexaenoic acid. A SwissADME analysis allows us to predict the pharmacokinetic properties of these small molecules. It assesses drug likeness and physicochemical properties, aiding drug discovery by evaluating potential interactions with biological systems [44]. Pharmacokinetic predictions revealed that those fatty acids exhibit strong lipophilic characteristics, influencing their solubility, permeability, and interaction with biological targets (Table 4). Oleic acid demonstrated moderate-to-poor aqueous solubility, with Log S values ranging from −5.4 (ESOL) to −8.3 (Ali), indicating variability across predictive models. It exhibited high gastrointestinal (GI) absorption but did not penetrate the blood–brain barrier (BBB), suggesting limited central nervous system activity [45]. Additionally, its inhibition of CYP1A2 and CYP2D6 suggests potential drug–drug interactions, warranting further evaluation [46]. Linoleic acid showed high lipophilicity (Log P = 5.5) and moderate-to-poor solubility (ESOL Log S = −8.6). Despite its favorable GI absorption and minimal interactions with cytochrome P450 enzymes, its high molecular flexibility (16 rotatable bonds) may contribute to non-specific binding, requiring further experimental validation [47,48]. DHA displayed the highest lipophilicity (Log P = 7.0) and poor aqueous solubility (ESOL Log S = −10.9), potentially leading to aggregation in aqueous environments. Its pharmacokinetic profile indicates low GI absorption and a lack of BBB penetration, limiting its systemic distribution. However, it exhibited minimal cytochrome P450 interactions, except for potential CYP1A2 inhibition [49,50].

**Figure 5:**
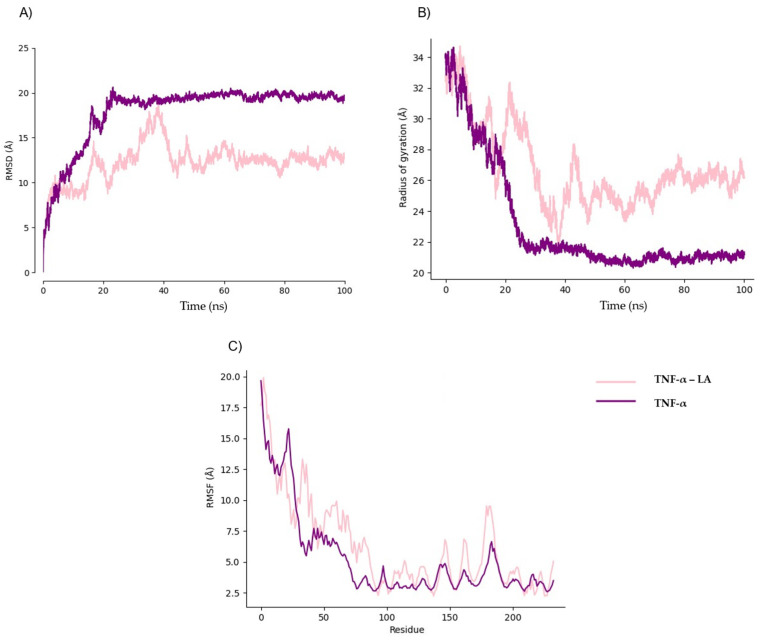
Comparative analysis of TNF-α structural dynamics in the presence and absence of linoleic acid. (**A**) Structural stability analysis (RMSD); (**B**) radius of gyration analysis; (**C**) fluctuation analysis (RMSF).

**Figure 6:**
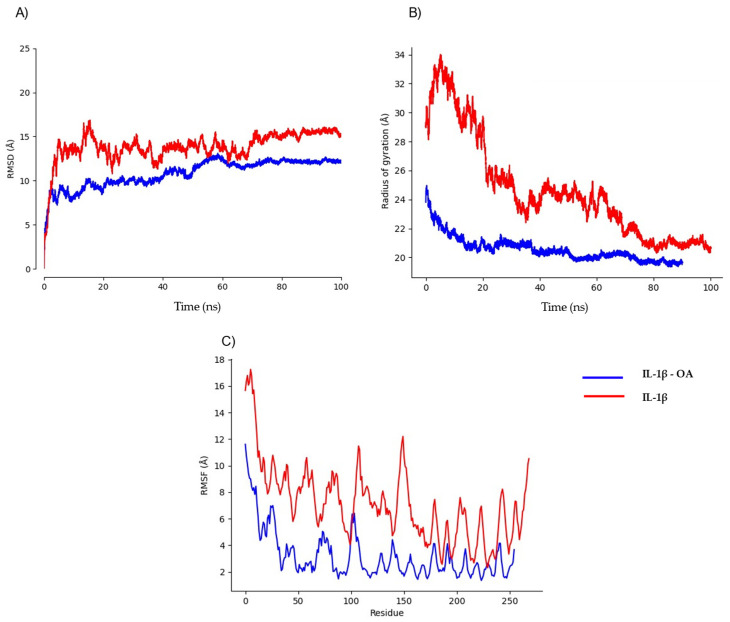
Comparative analysis of IL-1β structural dynamics in the presence and absence of oleic acid. (**A**) Structural stability analysis (RMSD); (**B**) radius of gyration analysis; (**C**) fluctuation analysis (RMSF).

**Figure 7:**
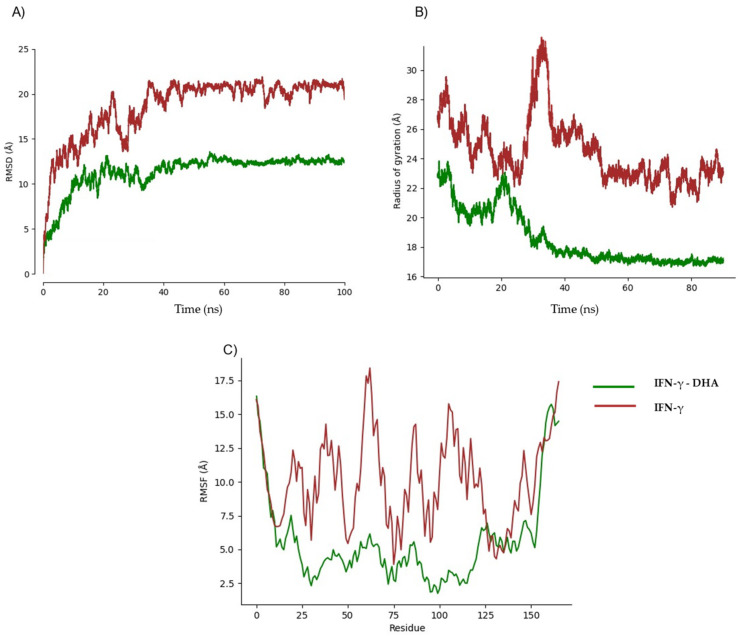
Comparative analysis of IFN-γ structural dynamics in the presence and absence of DHA. (**A**) Structural stability analysis (RMSD); (**B**) radius of gyration analysis; (**C**) fluctuation analysis (RMSF).

We performed MD simulations on protein structures modeled by in order to analyze their stability in the absence of ligands. These models exhibited significant fluctuations, prompting us to quantify their flexibility using MEDUSA software (Version 5.1.2). Our analysis revealed that TNF-α, IL-1β, and IFN-γ contain highly flexible regions, with over 50% of residues classified as disordered. To account for intrinsic flexibility, we conducted simulations both in the presence and absence of ligands to distinguishing ligand-induced conformational stabilization from natural structural fluctuations. Preliminary simulations determined that a 20 ns equilibration phase was necessary to achieve stability (Figure 5A, Figure 6A and Figure 7A). While experimentally determined crystal structures typically equilibrate within ~5 ns, AlphaFold models required an extended equilibration phase due to their dynamic nature and structural uncertainty (Figure 5B, Figure 6B and Figure 7B). Root mean square fluctuation (RMSF) analyses further supported these findings, showing that the highest fluctuations correspond to these disordered segments (Figure 5C, Figure 6C and Figure 7C), highlighting potential computational bias vs. true biological motion. While some flexibility may reflect real dynamics, further experimental validation is needed. Given this pronounced flexibility, MD simulations were adapted accordingly.

MD simulations were performed between TNF-α and linoleic acid. The RMSD showed a drastic change at 40 ns, increasing to 17.5 Ȧ, but then stabilizes at 60 ns, with the highest variation of 2 Ȧ (Figure 5A). A high fluctuation is observed in the gyration radius at 34 Ȧ decreasing at 60 ns, matching with the time of relatively stabilization in the RMSD (Figure 5B). The residues corresponding to the most flexible part of the protein (1–27, 56–87, 176–189) were the ones that showed the highest values of RMSF, with the total flexible region of the protein being 53%, as predicted with MEDUSA (Figure 5C).

When MD simulations were performed between IL-1β and oleic acid, the highest value of RMSD reached was at 12 Ȧ (Figure 6A). For IL1-β in a complex with oleic acid, although it showed strong interactions and higher stability, MD simulations revealed that this interaction was transient rather than stable over extended periods (Figure 6B). The radius of gyration showed large fluctuations in the first 50 ns (Figure 6C), but they eventually decreased and maintained optimal stability until the end of the simulation.

MD with IFN-γ and DHA showed average RMSD values of less than 14 Ȧ (Figure 7A), observing the most stable behavior, starting at 40 ns, where in turn, a decrease was seen. The fluctuations observed in the RMSF analysis corresponded to the most flexible sections, previously analyzed, in the first and last 20 residues of the structure (Figure 7B). The stabilization of the radius of gyration values was observed at 40 ns (Figure 7C), showing a behavior that suggests a stable complex, since ligands continue after 100 ns simulation.

To further investigate the conformational dynamics—structural stability, flexibility, and functional hotspots—relevant to ligand interactions of IL-1β, TNF-α, and IFN-γ during MD simulations, we performed principal component analysis (PCA) and Pearson cross-correlation analysis.

**Figure 8:**
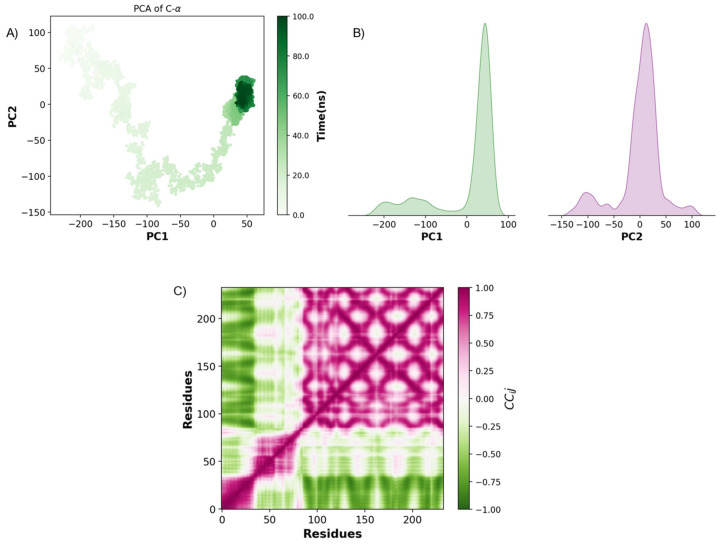
PCA and Pearson cross-correlation analysis of TNF-α dynamics. (**A**) PCA of TNF-α Cα atoms over 100 ns of molecular dynamics simulation. (**B**) Density plots of PC1 and PC2 components. (**C**) Pearson cross-correlation matrix (*CC_ij_*).

For TNF-α and linoleic acid MD simulated interaction, it exhibited substantial conformational flexibility in early simulation stages. Around 60 ns, its conformations converged, suggesting a transition to stable, functionally relevant states (Figure 8A). PCA density plots confirm this transition, showing a dominant peak in both principal components, indicating that the system reaches a preferred conformational state, potentially corresponding to functional binding interfaces (Figure 8B). Pearson cross-correlation analysis further supports this finding, identifying strongly coordinated motions and anti-correlated movements, highlighting structurally stable regions and flexible domains (Figure 8C). The presence of anti-correlated areas suggests potential allosteric sites that could influence TNF-α function.

**Figure 9:**
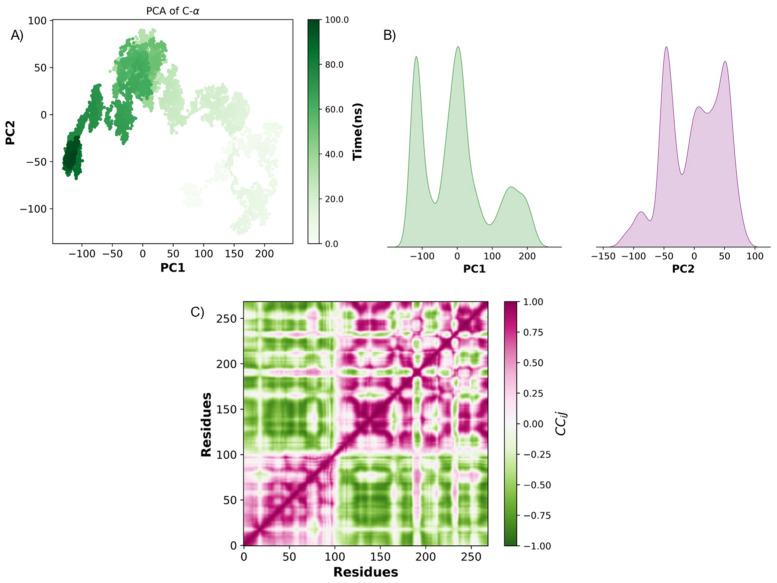
PCA and Pearson cross-correlation analysis of IL-1β dynamics. (**A**) PCA of IL-1β Cα atoms over 100 ns of molecular dynamics simulation. (**B**) Density plots of PC1 and PC2 components. (**C**) Pearson cross-correlation matrix (*CC_ij_*).

In the IL-1β and oleic acid PCA results, they showed wide conformational dispersion along PC1 and PC2 during the first 50 ns, indicative of high flexibility and conformational search (Figure 9A). However, after this period, the clustering of conformations was observed, suggesting structural stabilization into energetically favorable states. The density plots (Figure 9B) highlight the existence of multiple stable states, as indicated by distinct peaks in the PC1 and PC2 distributions. Pearson cross-correlation analysis shows regions with significant coordinated and anti-correlated motions (Figure 9C). Positively correlated regions corresponded to structurally stable domains, whereas negatively correlated regions highlighted flexible hinge-like movements, possibly involved in ligand-induced conformational changes. Notably, stabilization correlated with persistent interactions at TRP108, GLN164, and ILE103, suggesting that these residues serve as key interaction hotspots for ligand binding.

**Figure 10:**
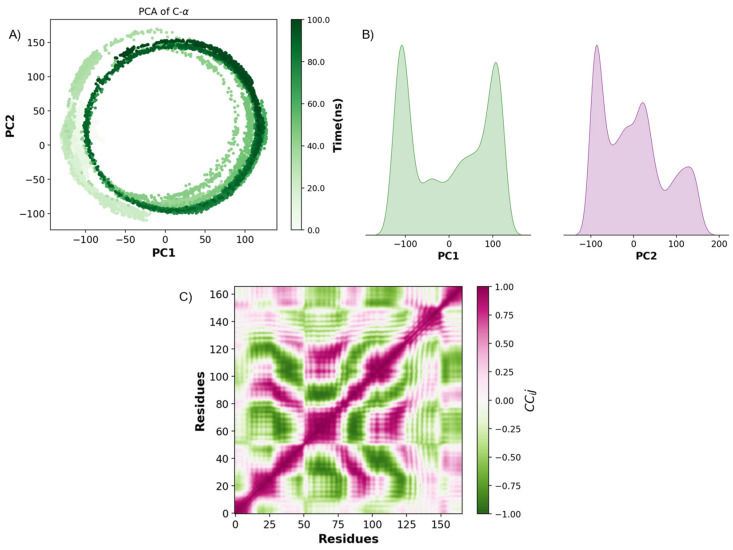
PCA and Pearson cross-correlation analysis of IFN-γ dynamics. (**A**) PCA of IFN-γ Cα atoms over 100 ns of molecular dynamics simulation. (**B**) Density plots of PC1 and PC2 components. (**C**) Pearson cross-correlation matrix (*CC_ij_*).

For IFN-γ and DHA, exhibits a distinct circular trajectory, indicative of periodic or cyclic conformational transitions between conformational states rather than complete stabilization. This pattern indicates intrinsic flexibility, particularly in the N- and C-terminal regions, allowing the cytokine to explore multiple states dynamically (Figure 10A). The density plots (Figure 10B) reflect multiple conformational ensembles, with peaks in PC1 and PC2 distributions suggesting transitions between these states. Pearson cross-correlation analysis further confirmed structural stable domains and dynamic regions with inverse movements (Figure 10C). These flexible regions could play a role in ligand binding-induced conformational shifts, influencing IFN-γ’s receptor engagement and downstream signaling.

Trajectory analysis using Visual Molecular Dynamics (VMD) further confirmed that ligand binding induced conformational changes, particularly in the flexible loop regions of IFN-γ. After 60 ns of MD simulations, these loop regions displayed increased mobility, suggesting that ligand interactions influenced the protein’s dynamic behavior. Similar structural adjustments were observed in TNF-α and IL-1β, where noticeable flexibility changes emerged after 60 ns, potentially affecting their allosteric regulation and interactions with downstream signaling partners. These structural adaptations could have functional implications for cytokine activity, offering valuable perspectives for future experimental validation and therapeutic targeting in inflammatory diseases such as atherosclerosis.

## 3. Discussion

Given the limitations of current therapies, an integrative approach targeting multiple cytokines represents a promising strategy for treating atherosclerosis. In this study, using an in silico drug-repositioning approach, we identified potential inhibitors of pro-atherogenic cytokines. However, these computational findings require in vitro and in vivo validation to confirm their biological activity and therapeutic potential. Our virtual screening and MD simulations identified molecules with documented cardiovascular benefits, such as inositol nicotinate, which formed three hydrogen bonds with ARG179 in the docking analysis. While these computational interactions suggest potential inhibitory effects, experimental validation is necessary to determine their actual biological activity. Inositol nicotinate, a niacin ester and vasodilator, plays an essential role in metabolic processes and is used as a lipid-lowering agent [51].

Fatty acids exhibited high docking scores against TNF-α, IL-1β, and IFN-γ. The cardioprotective properties of polyunsaturated fatty acids (PUFAs), particularly their roles in reducing LDL and triglycerides while increasing HDL, have been extensively reported in preclinical studies [40]. However, the molecular mechanisms underlying these effects remain poorly understood. Given their strong in silico interactions with cytokines, MD simulations were conducted to evaluate their binding stability. Despite these insights, in vitro and in vivo studies are essential to establish their actual therapeutic impact.

The Mediterranean diet, widely recognized for its antioxidant, anti-inflammatory, and immunomodulatory properties, has demonstrated significant benefits in preventing cardiovascular diseases, stroke, cognitive decline, and Alzheimer’s disease [41,42,43]. Fatty acids, which are key components of this diet, may interact with pro-atherosclerotic cytokines, potentially reducing foam-cell formation and modulate the inflammatory response. These interactions highlight the therapeutic potential of diet-derived compounds in mitigating atherosclerosis progression.

For TNF-α, linoleic acid presented 3 hydrogen bonding interactions at ARG179 and THR181 and 2 ionic bonds at residue ARG179. It has been reported that a higher intake of linoleic acid (bases on dietary surveys or biomarkers) is associated with a lower risk of mortality from cardiovascular diseases and cancer [52]. Also, the consumption of polyunsaturated fatty acids, specifically omega-3, such as eicosapentaenoic acid and DHA, results in low levels of plasma cholesterol and minimal coronary heart disease [53,54,55]. During the 100 ns MD simulation, the TNF-α-linoleic acid complex remained stable, with the ligand undergoing minor rearrangements. The peak at 17.5 Ȧ in ligand–protein interactions became more stabilized over time. After ~60 ns, the complex achieved relative structural stability, with a higher number of persistent residue interactions. While epidemiological evidence supports the cardioprotective effects of linoleic acid and omega-3 fatty acids, our computational findings suggest stable cytokine interactions, requiring further experimental validation to elucidate precise mechanisms of action.

DHA presented a high affinity for IFN-γ, showing 2 hydrogen bonds and 2 ionic bonds in LYS78. These findings align with reports that long-term benefits of polyunsaturated fatty acid intake in reducing the risk of cardiovascular disease and premature death [56]. The interactions that were maintained throughout the simulation with IFN-γ and DHA correspond to the residues PHE52 GLN71, ILE72 and PHE75. PHE52 is part of the hydrophobic surface that forms a small pocket at the protein interface, and together with LEU28 and TYR53 is involved in stabilizing the typical folding of IFN-γ [57], as well as in exposure for interaction with HSP70-like chaperones, such as DnaK [58], which mediates folding through ATP-dependent interactions with short linear peptide segments that are exposed in unfolded proteins. The observation of the interaction with PHE52 may be relevant, as it could affect the stability and folding of IFN-γ. Our in silico results indicate that DHA interacts with PHE52, a residue integral to IFN-γ stability and folding, suggesting a potential influence on cytokine structural dynamics. However, these predictions must be validated experimentally to confirm whether DHA affects IFN-γ stability and function in biological systems. This interaction is particularly significant given PHE52’s role in maintaining proper protein conformation and its involvement in chaperone-mediated folding processes [57]. IFN-γ’s high flexibility at terminal regions aligns with nuclear magnetic resonance studies demonstrating conformational shifts upon receptor binding [59]. The interaction of DHA with PHE52, identified in our simulations, suggests that fatty acids may influence IFN-γ stability. This contrasts with monoclonal antibodies like Fontolizumab, which target IFN-γ by blocking its receptor interaction [60,61]. The observed conformational shifts in PCA suggest that small molecules may offer a complementary mechanism to monoclonal antibodies by modulating IFN-γ’s structural flexibility rather than directly blocking its function. Further biochemical and biophysical studies are needed to confirm whether DHA can modulate IFN-γ activity through this mechanism. When considered alongside the well-documented cardiovascular benefits of polyunsaturated fatty acids, these findings further highlight the therapeutic potential of DHA in attenuating inflammation and contributing to the management of atherosclerosis.

Oleic acid presented 3 hydrogen bonds with IL-1β and 4 ionic bonds during virtual screening. It has been suggested that oleic acid could have antiatherogenic properties through a previous comparison of cholesterol metabolism in hamsters using dietary oleic acid and palmitic acids [62]. Epigenetically, hypermethylation patterns have been observed in atherosclerotic portions of the aorta, where an increase in DNA methylation has been noted with the progression of cardiovascular lesions [63,64,65]. Previous in vitro studies have shown that fatty acids can influence DNA methylation patterns. In our in silico analysis, oleic acid demonstrated stable interactions with IL-1β, particularly through TRP108, GLN164, and ILE103. Further biological experiments are required to confirm whether these interactions translate into functional cytokine modulation. Palmitic acid and arachidonic acid cause increases in DNA methylation in different cell types, while the effects of oleic acid are smaller and in the opposite direction, showing on the other hand a tendency towards hypomethylation [66]. Although IL1-β in complex with oleic acid showed strong interactions and higher stability, it does not interact with ARG120 during MD simulations. MD simulations initially predicted an interaction between oleic acid and ARG120, observed in docking studies, which was not sustained beyond the early stages of simulation, particularly after 100 ns. While virtual screening suggested strong initial binding, molecular dynamic simulations revealed that this interaction was transient rather than stable over extended periods. However, oleic acid consistently maintained stable interactions with TRP108, GLN164, and ILE103 throughout the simulation, suggesting alternative binding modes with potential functional relevance. Notably, TRP108 is the only tryptophan residue in the N-terminal region of pro-IL-1β, a site previously implicated in protease recognition due to its aromatic nature [38]. Given that IL-1β activation requires proteolytic cleavage, the stable interaction of oleic acid with TRP108 may modulate protease accessibility, potentially influencing IL-1β processing and activation. This observation aligns with structural studies on IL-1β inhibitors, such as Anakinra and Canakinumab, which block IL-1β signaling by preventing its interaction with IL-1R [67]. While these biologics function through receptor blockade, our findings suggest that small molecules like fatty acids may modulate IL-1β activation indirectly by altering protease accessibility and conformational dynamics. The residues that interacted during the entire simulation time corresponded to TRP108, GLN164, and ILE103; therefore, they had the most stable interactions. Of these residues, TRP108 is the only tryptophan residue in the N-terminal region of pro-IL-1β. The exposure of TRP108 and its specific recognition by enzymes such as chymotrypsin, which recognizes aromatic residues, could imply that this area could be susceptible to protease–substrate interactions [38]. These interactions may regulate the activity of pro-IL-1β by the action of proteases that cut specific sites to convert it to its active IL-1β form. Furthermore, the documented antiatherogenic properties of oleic acid, along with its association with hypomethylation patterns observed in atherosclerotic lesions, underscore its potential as a targeted therapeutic agent in the management and progression of atherosclerosis [66]. These findings suggest that oleic acid may exert its effects through non-canonical binding interactions, which could impact IL-1β function beyond direct cytokine inhibition. Further in vitro and in vivo studies will be necessary to validate these interactions and clarify their potential therapeutic implications in cytokine regulation.

The strong lipophilic properties of these fatty acids facilitate interactions with hydrophobic protein regions, making them promising candidates for modulating pro-inflammatory cytokines such as TNF-α, IL-1β, and IFN-γ [68,69]. However, their poor aqueous solubility and high molecular flexibility present challenges, including potential aggregation and non-specific interactions. Structural modifications or ligand optimization strategies may improve their binding specificity and pharmacokinetic profiles [70]. Despite violating some drug-likeness criteria, all three fatty acids remain viable for further study due to their stable molecular structures and previously reported biological activities. While our in silico findings highlight the potential of fatty acids as cytokine inhibitors, in vitro and in vivo validation are crucial to confirm their therapeutic viability. These findings underscore the importance of balancing lipophilicity and solubility to enhance their therapeutic potential in atherosclerosis management. Future work should prioritize experimental validation, pharmacokinetic assessments, and potential structural modifications to optimize their bioactivity and specificity [71].

Current therapeutic strategies for atherosclerosis and other inflammatory diseases often rely on monoclonal antibody therapies, which selectively neutralize pro-inflammatory cytokines. Examples include TNF-α inhibitors such as Infliximab, Adalimumab, and Etanercept, as well as IL-1β inhibitors like Canakinumab and Anakinra [10,72]. While these biologics have demonstrated clinical efficacy, they present several challenges, including high production costs, immunogenicity risks, and the need for parenteral administration [73]. However, structural studies have identified alternative allosteric binding sites, which could be targeted by small molecules [74]. Our PCA and cross-correlation analyses indicate that TNF-α transitions into a stable conformational state after 60 ns, revealing potential allosteric sites that may modulate TNF-α function. Unlike monoclonal antibodies, small molecules could target these sites to alter TNF-α signaling without direct receptor inhibition. These findings suggest an alternative mechanism for small-molecule modulation of TNF-α that requires further investigation in experimental settings. Additionally, the single-cytokine inhibition approach has limitations, as inflammatory pathways often involve compensatory mechanisms that can bypass the blockade of individual targets [75].

In contrast, our in silico approach explores small-molecule inhibitors capable of modulating multiple cytokines simultaneously, offering a potentially cost-effective and orally bioavailable alternative. Our virtual screening and molecular dynamics simulations identified fatty acids such as oleic acid, linoleic acid, and DHA as promising candidates, demonstrating stable interactions (in silico) with TNF-α, IL-1β, and IFN-γ. Unlike monoclonal antibodies, these small molecules may exert broader regulatory effects on cytokine networks without the risk of inducing an anti-drug immune response. Moreover, some of these compounds, particularly polyunsaturated fatty acids, have well-documented cardiovascular benefits and a favorable safety profile [71,76].

However, several challenges remain in translating our findings into viable therapeutics. The efficacy of small-molecule inhibitors in cytokine targeting is less established than that of monoclonal antibodies, and their pharmacokinetics, specificity, and bioavailability require further optimization. While our study demonstrates stable cytokine–ligand interactions in silico, additional in vitro and in vivo validation is crucial to assess their biological activity, metabolic stability, and potential off-target effects.

Ultimately, our approach complements rather than replaces existing cytokine-targeting therapies. Combining small molecules with monoclonal antibodies or exploring synergistic effects between these therapeutic classes may represent an innovative strategy for improving treatment outcomes in inflammatory diseases. Further research is needed to establish the comparative therapeutic potential of these small molecules and determine their feasibility as standalone or adjunct therapies.

## 4. Materials and Methods

Molecular modeling, electric partial-charge assignation, ligand conformer, searching of potential binding sites, energy minimizations, visualization and docking were performed with Molecular Operating Environment (MOE), v2020.09, Chemical Computing Group ULC, Montreal, QC, Canada. [77].

### 4.1. Ligand Preparation

The chemical structure of 2750 ligands that comprise the updated list of FDA-approved drugs were obtained from the DrugBank database [78]. To facilitate ligand flexibility in our rigid docking simulations, we utilized the Conformer Import tool (MOE) to generate a series of low-energy conformers for each drug. Our approach imposed a conformational cut-off energy of 3 kcal/mol from the minimum energy structure of each compound, as calculated with the AMBER10-EHT force field. This resulted to an in-house molecular database that includes multiple conformers for each molecule, which were then employed in our rigid docking simulations.

### 4.2. Protein Selection for Ligand Docking

The predicted protein structures of TNF-α (AF-P01375-F1), pro-IL-1β (AF-P01584-F1), and IFN-γ (AF-P01579-F1) (Figure 1) were retrieved from the AlphaFold database and selected as target proteins for MD simulations. AlphaFold’s AI-modeled proteins complement experimental methods by providing highly accurate structural predictions, reducing time and costs, and enabling rapid hypothesis generation. These models facilitate interaction analysis, mutation exploration, and experimental data refinement, enhancing insights into protein function and structural complexes [29]. To assess the reliability of the predicted structures, pLDDT scores were analyzed. pLDDT is a per-residue confidence metric (0–100), where scores > 90 indicate high accuracy for both backbone and side chains, while scores > 70 suggest a well-modeled backbone with possible side-chain deviations. Variability in pLDDT values across a protein highlights regions of higher or lower confidence, guiding the interpretation and reliability of the predicted models [79,80].

Hydrogen atoms (Protonate 3D tool) and partial charges (Potential Setup tool) were added to TNF-α, IL-1β, and IFN-γ assuming a pH equal to 7.0 and using the AMMBER-10-EHT forcefield, respectively. The later, potential binding sites in TNF-α, IL-1β, and IFN-γ, containing residues previously reported to be associated with receptor binding sites, were identified using the SiteFinder tool. Prior to each docking, the three structures were subjected to energy minimization using the same forcefield, to optimize atomic contacts. Docking simulations between each of the optimized TNF-α, IL-1β and IFN-γ structures and each of the conformers contained in the in-house database were carried out under the rigid-docking protocol. The docking parameters were set to take each ligand conformation as a unique molecule, using the Alpha Triangle algorithm as the placement method—at least 200 different orientations or poses on a potential binding site—and a further evaluation, keeping the thirty best poses; accordingly, the London scoring function for binding affinity with a second refinement as a Rigid Receptor using the Affinity dG algorithm was used, keeping the ten best potential binding poses. Further refinement was made by induced fit, allowing for conformation flexibility in the ligand and receptor side chains. The results were analyzed by docking score, the frequency of the chemical compound as a stable conformation, and the types of interactions at the binding-site residues.

### 4.3. Pharmacokinetic and Drug-Likeness Prediction

The pharmacokinetic behavior and drug likeness of the selected compounds was assessed using SwissADME (www.swissadme.ch/citing.php, accessed on 20 March 2025) [44]. The 2D structures of the fatty acids were obtained and converted into Canonical SMILES, which were then uploaded to the SwissADME platform. The input canonical SMILES were as follows: oleic acid, CCCCCCCC/C=C/CCCCCCCC(=O)O; linoleic acid, CCCCCCCCCCCCCCCCCC(O)O; DHA, CCCCCCCCCCCCCCCCCCCCCC(O)O. SwissADME generated ADMET predictions, including lipophilicity (Log P), solubility (Log S), gastrointestinal absorption, blood–brain barrier permeability, cytochrome P450 interactions, and drug-likeness assessments based on Lipinski’s rule of five and other filters. In this analysis, Log P values indicate hydrophilicity (negative value) or lipophilicity (positive values), influencing absorption and metabolism. Log S (ESOL and Ali) values were used to estimate solubility, where higher values indicating greater solubility, while lower values suggest poor aqueous solubility.

### 4.4. Molecular Dynamics Simulations

We conducted MD simulation studies using the same predicted protein structures of TNF-α, IL-1β, and IFN-γ, employing the OpenMM toolkit within the Google Colab framework through a Jupyter Notebook environment [81]. The protein topology of each structure was generated using the Amber ff19SB forcefield, while the General Amber Force Field 2 (GAFF2) was employed for the ligand to ensure compatibility with the protein forcefield. The simulation box was constructed using the TIP3P water model to simulate the solvent environment, with a box size of 12 Å, ensuring sufficient spacing between the solute and the box edges to minimize boundary effects. To simulate physiological conditions, a concentration of 0.15 M NaCl was added, and the system was automatically neutralized using AMBER’s leap tool. The system was energy-minimized for 50,000 steps using the steepest descent algorithm to remove steric clashes and stabilize the initial conformation. Equilibration was performed under isothermal–isobaric (NPT) conditions at 310 K using a Langevin thermostat with a collision frequency of 1 ps^−1^ and a Monte Carlo barostat set to 1 atm. A time step of 2 fs was applied, and hydrogen bonds were constrained. Production runs of 100 ns were conducted using the OpenMM toolkit within the Google Colab framework through a Jupyter Notebook environment. Structural stability was assessed through the calculation of root mean square deviation (RMSD) and root mean square fluctuation (RMSF). Conformational changes were monitored by analyzing the radius of gyration. Trajectory analysis was performed using VMD software (version 1.9.4) to evaluate the structural stability and conformational dynamics of the protein–ligand complexes [82].

### 4.5. Protein Flexibility Predictions

Highly deformable regions and the dynamic properties of the proteins were analyzed using MEDUSA software (Version 5.1.2) [83], which specializes in predicting intrinsic flexibility and structural adaptability. MEDUSA assigns a flexibility class to each position in the protein sequence based on a comprehensive evaluation of local structural features, sequence characteristics, and dynamic behavior. This classification provides valuable insights into the region most likely to undergo conformational changes, which are critical for functions such as ligand binding, protein–protein interactions, and allosteric regulation.

### 4.6. Principal Component Analysis (PCA)

To analyze large-scale conformational changes, PCA was performed on the MD trajectories using the covariance matrix of Cα atomic fluctuations for TNF-α-linoleic acid, IL-1β-oleic acid and IFN-γ-DHA. The first two principal components (PC1 and PC2) were extracted and visualized to identify dominant conformational states. The trajectories were first centered and fitted to remove rotational and translational motions. Later, the atomic fluctuations of backbone Cα atoms were computed, and an eigenvalue decomposition was applied to extract the principal components. Finally, PCA plots were generated, where PC1 vs. PC2 scatter plots illustrate the sampled conformational space over time, and PC density plots reveal the most populated conformational states.

### 4.7. Dynamic Cross-Correlation Analysis

To assess residue–residue motion relationships, cross-correlation analysis was performed using the Cα displacement covariance matrix. The correlation coefficient (*C_ij_*) between residues *i* and *j* was calculated using:

(1) $$C_{ij}=\frac{\left(∆r_{i}\cdot ∆r_{j}\right)}{\surd (∆r_{i}^{2})\cdot (∆r_{j}^{2})}$$

where Δ*r* represents atomic displacement over time in the MD trajectory. The generated matrix was generated in the Google Colab framework. A positive correlation (*CC_ij_* > 0) signifies that two residues move in a synchronized manner, while a negative correlation (*CC_ij_* < 0) indicates opposing directional movements. A strong correlation suggests that the residues exhibit coordinated motion with the same phase and frequency. Conversely, values deviating from 1 or −1 imply weaker correlations, meaning the movements of residues *i* and *j* are less synchronized, or exhibit reduced anti-correlation.

## 5. Conclusions

An in silico drug-repositioning strategy was conducted on atherogenic cytokines, TNF-α, IL-1β and IFN-γ, using protein structures predicted by AlphaFold. A virtual screening approach, using a library of FDA-approved drugs, identified several compounds with lipid-lowering properties, highlighting their potential therapeutic utility in the treatment of atherosclerosis. Among these, fatty acids such as DHA, linoleic acid, and oleic acid were of particular interest due to their well-documented nutraceutical and antiatherogenic properties. Additional compounds, such as inositol nicotinate, also demonstrated promising interactions. MD simulations of those atherogenic cytokines with fatty acids revealed stable interactions, after 100 ns, with key cytokine residues involved in critical biological processes. Furthermore, our study provides insights into the structural dynamics of IL-1β, TNF-α, and IFN-γ, identifying key interaction hotspots and potential allosteric regulatory sites. Unlike monoclonal antibody therapies, which primarily function through receptor blockade, our findings suggest that fatty acids may modulate cytokine activity by influencing structural flexibility and the accessibility of key functional residues. This highlights their potential as modulators of cytokine function through indirect structural effects rather than direct inhibition. Notably, fatty acids exhibited strong binding affinities and maintained consistent interactions with residues essential for cytokine function. These interactions provided insights into their potential to modulate cytokine activity and influence atherosclerosis progression. While these findings present a novel perspective on cytokine-targeting strategies, in vitro and in vivo validation remains essential to confirm these interactions and their therapeutic implications. Future studies should integrate experimental binding assays, functional enzymatic studies, and mutagenesis experiments to assess whether these computationally identified sites significantly impact cytokine activity regulation. By analyzing these interactions at in silico level, our findings underscore the need for further investigation to determine whether small-molecule cytokine inhibitors can serve as standalone or adjunct therapies in inflammatory disease management. Our findings could facilitate the design of focused and highly effective therapeutic interventions. Additionally, expanding this approach to other inflammatory cytokines and small-molecule inhibitors may further elucidate the mechanistic advantages and therapeutic feasibility of targeting cytokine structural dynamics as an alternative to monoclonal antibody therapies. This integrative strategy facilitates the development of novel therapeutics for inflammatory diseases beyond atherosclerosis.

## Figures and Tables

**Table 1:** Key structural characteristics for potential binding sites for TNF-α and IL1-βγ.

Protein	Binding Site	Structural Characteristics	Functional Implications
TNF-α	Residues 178–180 (E-F loop, core epitope)	Located in the E-F loop; part of the monomer that forms a pore through the trimer’s center.	Critical for structural stability and epitope functionality [31,32,33].
IFN-γ	Residue LEU136 (with GLU135)	Found in a region involved in truncation affecting the C-terminal; high confidence (pLDDT > 90).	Key in reducing JAK-STAT1 signaling and pro-inflammatory macrophage activation [35].
IL-1β	Residue ARG120 (N-terminal). Residues in the C-terminal core region	Stabilizes the tertiary structure and receptor-binding domain; part of the pro-IL-1β precursor. Becomes part of the functional mature IL-1β.	Essential for maintaining protein stability and facilitating receptor interaction. Critical for biological activity and proteolytic maturation by Caspase-1 [36,37,38].

**Table 2:** Selected union sites for TNF-α, IL1-β and IFN-γ.

Protein	Size	PLB	Hyd.	Side	Residues
TNF-α	30	1.78	14	22	GLY144 CYS145 PRO176 CYS177 GLN178 ARG179 GLU180 THR181 ALA185 GLU186 ALA187 LYS188 TRP190
IL-1β	84	1.38	42	63	SER66 VAL67 PHE94 PHE98 GLU99 GLU100 GLU101 PRO102 ILE103 PHE105 TRP108 TYR113 VAL114 HIS115 VAL119 ARG120 PHE162 VAL163 GLN164 GLU167 PRO173 LYS209 LYS210
IFN-γ	32	0.86	21	23	SER74 LYS78 PHE115 LEU118 TYR121 SER122 VAL123 ALA132 ILE133 LEU136

The size column indicates the number of residues in the pocket. The PLB column indicates the protein–ligand binding index. The Hyd column indicates the number of hydrophobic contact atoms in the receptor. The side column indicates the number of sidechains in the pocket. Key structural residues are highlighted in black.

**Table 3:** Binding scores and interaction profiles of selected ligands with TNF-α, IL-1β and INF-γ in molecular docking analysis.

TNF-α								
Ligand Name	Freq	S-Score	Interaction Type					
			H-Bond	H-Bond Residue Interaction	I-Bond	I-Bond Residue Interaction	Pi-Bond	Pi-Bond Residue Interaction
carisoprodol	2000	−4.5	5	CYS145, PRO176, THR181, ARG179 (2 interactions)	0	-	0	-
acebutolol	2000	−4.7	4	PRO176, THR181, THR181, ARG179	0	-	0	-
atracuryum becilate	2000	−6.7	3	THR181, TYR191, GLU192	0	-	1	PHE140
inositol nicotinate	2000	−6.7	3	ARG179 (2 interactions), THR181	0	-	0	-
docusate	2000	−6.1	3	ARG179 (2interactions),THR181	2	ARG179 (2 interactions)	1	TRP190
lauryl sodium sulfoacetate	2000	−5.1	3	GLU180 (2 interactions), THR181	0	-	0	-
palmitic acid	2000	−4.8	3	CYS145,THR181, ARG179	2	ARG179 (2 interactions)	0	-
linoleic acid	2000	−4.4	3	ARG179 (2 interactions), THR181	2	ARG179 (2 interactions)	0	-
lauric acid	2000	−4.3	3	THR181, ARG179 (2 interactions)	2	ARG179 (2 interactions)	0	-
cetil alcohol	2000	−4.3	2	GLU180, THR181	0	-	0	-
IL-1β								
tetradecyl hydrogensulfate (ester)	1969	−6.3	5	LYS209, SER66, VAL67, ARG120 (2 interactions)	4	ARG120 (4 interactions)	1	PHE98
sodium tetradecyl sulfate	1890	−6.1	5	SER66, VAL67, ARG120 (2 interactions), LYS209	4	ARG120 (4 interactions)	0	-
docusate	2000	−7.2	4	LYS209, ARG120 (2 interactions), VAL67	6	ARG120 (6 interactions)	0	-
carisoprodol	2000	−5.5	4	VAL67, GLU101, VAL67, ARG120	0	-	0	-
ioxilan	1840	−9.1	4	GLN164, GLN164, ARG120, VAL119	0	-	0	-
capecitabine	1680	−6.9	4	GLU101, LYS209, ARG120, VAL67	0	-	0	-
oleic acid	2000	−6.5	3	ARG120(3 interactions)	4	ARG120 (4 interactions)	0	-
stearic acid	2000	−5.7	4	ARG120 (3 interactions), LYS 209	4	ARG120 (4 interactions)	0	-
lutein	2000	−7.6	2	GLU101, ARG120	0	-	0	-
doconexent	2000	−7.2	2	ARG120 (2 interactions)	5	ARG120 (5 interactions)	0	-
IFN-γ								
sodium tetradecyl sulfate	2137	−11.4	3	LYS78, SER122, VAL123	1	LYS78	0	-
doconexent	2122	−9.6	2	LYS78 (2 interactions)	2	LYS78 (2 interactions)	0	-
acebutolol	2492	−9.8	3	SER122, SER74, LYS78	0	-	0	-
lauric acid	2117	−9.1	2	VAL123, LYS78	1	LYS78	0	-
capecitabine	2155	−10	2	SER122, LYS78	0	-	0	-
methocarbamol	1871	−8.7	2	LYS78 (2 interactions)	0	-	0	-
anisotropin methyl bromide	1688	−9.6	2	GLN129, LYS78	0	-	0	-
yofendilate	2102	−9.5	1	LYS78	0	-	1	VAL123
hexaminolevulinate	1725	−8.5	1	LYS78	0	-	0	-
carisoprodol	2830	−8.8	1	LYS78	0	-	0	-

**Table 4:** Molecular and physicochemical predictions of selected fatty acids.

Descriptor	Oleic Acid	Linoleic Acid	DHA
ESOL Log S	−5.4	−5.5	−7.0
ESOL Class	Moderately soluble	Moderately soluble	Poorly soluble
Ali Log S	−8.3	−8.6	−10.9
Ali Class	Poorly soluble	Poorly soluble	Insoluble
GI absorption	High	High	Low
BBB permeant	No	Yes	No
CYP1A2 inhibitor	Yes	Yes	Yes
CYP2C19 inhibitor	No	No	No
CYP2C9 inhibitor	Yes	No	No
CYP2D6 inhibitor	No	No	No
CYP3A4 inhibitor	No	No	No

## Data Availability

Data are contained within the article.

## Abbreviations

The following abbreviations are used in this manuscript:

TNF-α	Tumor necrosis factor-α
IL-1β	Interleukin-1β
IFN-γ	Interferon-γ
mRNA	Messenger RNA
AI	Artificial intelligence
FDA	Food and Drug Administration
pLDDT	predicted local distance difference test
DHA	docosahexaenoic acid
ns	Nanosecond
MD	Molecular dynamics
atm	Atmospheres
PCA	Principal Component Analysis
